# A Graph Convolutional Network Based on Univariate Neurodegeneration Biomarker for Alzheimer’s Disease Diagnosis

**DOI:** 10.1109/JTEHM.2023.3285723

**Published:** 2023-06-13

**Authors:** Zongshuai Qu, Tao Yao, Xinghui Liu, Gang Wang

**Affiliations:** School of Information and Electrical EngineeringLudong University 12405 Yantai 264025 China; Shandong Vheng Data Technology Company Ltd. Yantai 264003 China; School of Ulsan Ship and Ocean CollegeLudong University 12405 Yantai 264025 China

**Keywords:** Alzheimer’s disease, magnetic resonance imaging, univariate neurodegeneration biomarker, graph convolutional network, attention module

## Abstract

Objective: Alzheimer’s disease (AD) is a progressive and irreversible neurodegenerative disease that is not easily detectable in the early stage. This study proposed an efficient method of applying a graph convolutional network (GCN) on the early prediction of AD. Methods: We proposed a univariate neurodegeneration biomarker (UNB) based GCN semi-supervised classification framework. We generated UNB by comparing the similarity of individual morphological atrophy pattern and the atrophy pattern of $\text{A}\beta +$ AD group according to the brain morphological abnormalities induced by AD. For the GCN semi-supervised classification model, we took the UNBs of individuals as the features of nodes and constructed the weight of edges according to the similarity of phenotypic information between individuals, which explored the essential features of individuals through spectral graph convolution. The attention module was constructed and embedded into the GCN framework, which may refine the input morphological features to highlight the main impact of AD on the cerebral cortex and weaken the instability caused by individual diversities, thereby identifying the significant ROIs affected by AD and improving the classification accuracy. Results: We tested the UNB-GCN framework on the Alzheimer’s Disease Neuroimaging Initiative (ADNI) database. The estimated minimum sample sizes were 156, 349 and 423 for the longitudinal $\text{A}\beta +$ AD, $\text{A}\beta +$ mild cognitive impairment (MCI) and $\text{A}\beta +$ cognitively unimpaired (CU) groups, respectively. And the proposed UNB-GCN framework combined with the attention module can effectively improve the classification performance with 93.90% classification accuracy for AD vs. CU and 82.05% for AD vs. MCI on the validation set. Conclusion: The proposed UNB measures were superior to the conventional volume measures in describing the AD-induced cerebral cortex morphological changes. And the UNB-GCN framework combined with attention module may effectively improve the classification performance between MCI subjects and AD patients. Clinical and Translational Impact Statement: This study aims to predict the early AD patients, so as to help clinicians develop effective interventions to delay the deterioration of AD symptoms.

## I. Introduction

Alzheimer’s disease (AD) is a progressive and irreversible neurodegenerative disease which is expected to affect more than 100 million families by 2050 [1]. In order to maximize the efficacy of AD prevention therapies, the individuals at high risk of AD, including the cognitively unimpaired older adults (65 years) with accumulation of beta-amyloid plaques ( $\text{A}\beta +$) [2] and the carriers of the apolipoprotein E (APOE) 4 allele [3], need to be treated prior to measurable impairments in cognition, at which time the biomarkers associated with pathology have been developed [4]. The commonly used biomarkers related to AD include fluorooxyglucose positron emission tomography (FDG-PET) measurement of brain glucose metabolism rate [5], standard uptake value ratio (SUVR) of florbetapir PET about the changes in amyloid plaque burden [6] and magnetic resonance imaging (MRI) structural measurements of the brain morphological changes [7], [8]. Due to the close relationship between MRI biomarker and neurodegeneration, a major research in recent years has been using brain imaging biomarkers, including cortical atrophy [9], hippocampal atrophy [10], [11], and ventricular enlargement [12], for differential diagnosis and tracking of AD. By designing a diagnostic classification model based on the MRI biomarkers, we can effectively perceive the brain morphological changes of individuals affected by AD. And the patients with mild symptoms, such as mild cognitive impairment (MCI) defined as the early stage of AD, may be implemented to delay the progression of AD through the targeted interventions [13], [14].

With the development of machine learning, including support vector machines (SVM) [15], linear discriminant analysis (LDA) [16], decision trees [17] and artificial neural networks [18], which has been widely applied to the classification of AD and obtained relatively accurate classification results through statistical analysis on the extracted MRI biomarkers. However, due to the strong individual morphological differences and the inhomogeneity of dementia, there are some issues to be considered to improve the classification accuracy.

First, due to the high individual variability and the complex geometry of the cortical surface [19], it is major challenge to generate the essential MRI morphological features induced by AD with high statistical discrimination ability, which will improve the accuracy of the classification model. Second, due to the inhomogeneity of AD-induced cortical morphology changes and the excessive noise introduced during MRI acquisition, it is difficult for the classification framework based on traditional machine learning algorithms, i.e., SVM, LDA, Decision trees, etc., to obtain higher classification accuracy. In addition, the correlation of AD-induced morphological structures among individuals is not fully exploited in the classification mechanism, which also affects the discriminative ability of the classification model.

Based on the above discussion, we need to consider how to improve the accuracy of the classification model from two aspects, i.e., AD-induced morphological features and classification mechanism. Most of the previous research extracted the MRI cortical geometrical features on the predefined regions of interest (ROIs) which are formed by the statistical group difference analysis [20], [21]. However, these ROIs lack the guidance of the cortical anatomical information, which tends to generate the unreliable ROIs and is not conducive to revealing the relationship between the anatomical morphological abnormalities and AD-related symptoms.

To generate the reliable and robust ROIs, we combined the registration method and the anatomical information of the original cortical surfaces provided by FreeSurfer [22], [23] to re-establish the spatial and anatomical correspondences between the new registered individual surfaces. Then all the registered anatomical regions, referred as all the ROIs of the whole brain, can construct the overall AD-induced structural abnormality correspondences between the individuals by the guidance of anatomical information. On each obtained ROI, the univariate neurodegeneration biomarker (UNB) can be generated based on our previous work [24] for reliably and stably quantifying the morphological changes induced by AD. Finally, we can get a 1D feature vector for each individual containing n UNBs (i.e., AD-induced morphological features) computed over n ROIs (i.e., anatomical regions).

For classification mechanism, it is difficult to obtain high classification accuracy by directly applying traditional machine learning methods on the UNBs due to the large differences between the individual morphological changes caused by AD. Recently, deep learning methods have been developed to solve complicated problems in various fields [25], [26]. In particularly, convolutional neural network (CNN) [27] is widely applied in the fields of pattern recognition and computer vision, which can automatically learn the implicit features with rich meaning from the input data through convolution layers and can effectively improve the classification accuracy. However, the input data is limited within Euclidean-structure in CNN, which is difficult to exploit the correlation between the individuals. Thus, an efficient variant of CNN, referred as graph convolutional network (GCN) [28], was proposed to achieve very promising classification results through applying spectral graph theory on the non-Euclidean data structure that encodes both the features of nodes and the correlation between the nodes.

Due to the diversity of individual atrophy changes caused by AD [29], it is worth noting that if the weight of atrophy on each ROI is not identified, the computational complexity of the GCN classification model will increase and the classification accuracy will decrease. However, the popular GCN framework does not contain a discriminative module for each UNB defined on each ROI. To address this issue, we plan to embed an attention module in the GCN framework [30], [31], which is usually used as an insertion module during training to refine the input UNBs, which may make the network focus on important regions where the significant UNBs were generated. Based on the attention module, we may assign each UNB a weighting factor that represents the degree of the common statistical atrophy affected by AD. Through revealing the significant UNBs defined on the significant ROIs affected by AD, the attention module may improve the classification performance and decrease the computational complexity [31].

In this paper, we plan to construct an UNB-GCN semi-supervised classification framework incorporating an attention module to differentiate the MCI subjects and AD patients. The contributions can be summarized as follows:

1. The univariate neurodegeneration biomarker (UNB) generation method considering the anatomic information is proposed to comprehensively capture the AD-induced brain morphometry abnormalities and reveal the relationship between the anatomical morphological abnormalities and AD-related symptoms.
2. The graph convolutional network (GCN) framework is proposed, which may improve the classification accuracy by automatically learning not only the node features composed of the UNBs of individual, but also the association information between nodes based on the estimated similarity of phenotypic information between individuals.
3. The attention module is constructed and embedded into the GCN framework, which may refine the input morphological features to highlight the main impact of AD on the cerebral cortex and weaken the instability caused by individual diversities, thereby identifying the significant ROIs affected by AD and improving the classification accuracy.

We hypothesized that our proposed UNB-GCN classification framework may outperform traditional machine learning-based classification algorithms in early AD diagnose.

UNB-GCN’s source code and datasets are available at https://github.com/Zongshuaiqu/UNB-GCN.git.

## II. Material and Methods

### A. Subjects

Data is downloaded from the ADNI database [32]. ADNI is the result of efforts of many co-investigators from a broad range of academic institutions and private corporations. It enables researchers around the world to share data. Subjects have been recruited from over 50 sites across the U.S. and Canada. The primary goal of ADNI is to test whether biological markers, such as serial MRI and positron emission tomography (PET), combined with clinical and neuropsychological assessments, can measure the progression of mild cognitive impairment (MCI) and early AD. Subjects originally recruited for ADNI-1 and ADNI-GO had the option to be followed in ADNI-2. For up-to-date information, see https://www.adniinfo.org.

In this study, we used brain structural MRI data from 127 $\text{A}\beta +$ AD patients and 131 $\text{A}\beta $- cognitively unimpaired (CU) to identify the ROIs which were further used to define UNB. Because the studies have shown that the cerebral cortex of the left hemisphere shrinks faster than that of the right hemisphere under the influence of AD [33]. Therefore, we would focus our research on the left hemisphere of brain. To validate the statistical discrimination ability of UNB, we studied the longitudinal UNB changes through group differences and minimum sample size estimation. We used the 318 $\text{A}\beta +$ subjects, including 84 AD, 102 MCI, and 132 CU subjects to train the Attention-based UNB-GCN. To validate the classification performance of Attention-based UNB-GCN, we input 128 $\text{A}\beta +$ validation subjects which were at 24-months test, including 32 AD, 46 MCI, and 50 CU subjects into the graph convolutional network, to classify the AD vs. MCI and AD vs. CU.

### B. Image Acquisition

High-resolution brain structural MRI scans were acquired using 3 Tesla MRI scanners manufactured by General Electric Healthcare, Siemens Medical Solutions, and Philips Medical Systems. For each subject, a high-resolution T1 magnetization-prepared spoiled gradient (SPGR) scan was obtained in the sagittal plane. A T1-weighted pulse sequence (radiofrequency-SPGR recall acquisition in the steady state, repetition time = 33 msec, echo time = 5 msec, alpha = 30°, number of excitations = 1, field-of-view = 24 cm, imaging matrix = $256\times93$, slice thickness = 1.5 mm, scan time = 13:36 min) was used to acquire 124 congiguous horizontal MRI slices with in-plane voxel dimensions of $0.94\times1.25$ mm.

### C. Surface Registration

The MRIs of all subjects were preprocessed with automatic FreeSurfer pipeline [34], including skull stripping, B1 bias field correction, and gray/white matter segmentation. After the reconstruction of the cortical gray matter surface is completed, we used the spherical harmonic registration method [18] to establish the correspondence between different outer surfaces, and finally achieved the registration of different individuals. Moreover, FreeSurfer uses the Desikan/Killiany atlas partition method to label brain regions during image processing [35]. This partitioning method divided the cerebral cortex of left hemisphere into 34 anatomical brain regions. We called each anatomical partition a candidate ROI. According to the anatomical partition information of FreeSurfer, there were a total of 34 candidate ROIs in the left hemisphere.

### D. Univariate Neurodegeneration Biomarker (UNB)

We extracted the thickness measurements of all ROIs from 127 $\text{A}\beta +$ AD subjects and 131 $\text{A}\beta $- CU subjects. Considering the influence of three factors such as age, gender and group among subjects, we used general linear model in the SurfStat software package (https://www.math.mcgill.ca/keith/surfstat) to obtain the intrinsic thickness measurements on each vertex of all the ROIs. Next, we computed the group mean thickness difference vertex by vertex between the AD group and the CU group to obtain the $t$-score of each vertex on the ROIs. After transforming the $t$-scores to the $z$-scores for AD group, we obtained the AD group atrophy pattern. For the individual atrophy pattern, we selected 318 $\text{A}\beta +$ subjects, including 84 AD, 102 MCI, and 132 CU subjects. We computed the group difference and the $t$-score vertex by vertex on the ROIs between the thickness of individual subject and the average thickness of the CU group including 131 $\text{A}\beta $- CU subjects. We transformed the $t$-scores of each vertex to the $z$-scores to obtain the atrophy pattern of the testing individual. Finally, UNB of each ROI can be obtained by comparing the similarity between the individual atrophy patterns with the AD group atrophy pattern on the predefined ROI:

\begin{equation*} UNB_{k}=\frac {\sum \limits _{i=1}^{m_{k}} {Z_{sk_{i}} \cdot Z_{k_{i}}}}{100} \tag{1}\end{equation*}

where $k$ is the serial number of the ROI, $N=C\frac {\sigma ^{2}}{(m-b)^{2}}$ is the $z$-score of $i$th vertex in the $k$th ROI for the testing individual which is called the individual atrophy degree, and $Z_{sk_{i}}$ is the $z$-score of $i$th vertex in the $k$th ROI for AD group which is called AD group atrophy degree. From Eq. (1), we could see that the UNB measured how similar the individual atrophy degree was when compared to the AD group atrophy degree in the selected ROI. The greater the UNBs were, the closer the individual atrophy degree from the AD morphological characteristics. So we obtained a feature vector consisting of 34 UNBs describing the atrophy degree of the left hemisphere of an individual.

### E. UNB-GCN Framework With Attention Module

The goal of UNB-GCN model is to learn a function $f(\textit {X, W})$ on a graph ${G} = \{V, E, W\}$, which takes $G$ as input and produces a node-level output $Z$. And $V$, $E$ and $W$ represent nodes, edges, and adjacency matrix of the graph, respectively. And every feature vector $x(i,\bullet)$ for every node $i$, i.e., the UNBs of $i$-th subject, is summarized into a feature matrix $X\in R^{N\times M}$, where $N$ is the number of nodes and $M$ is the feature dimension. The UNB-GCN framework contains $K$ neural network layers with the following layer-wise propagation rule:

\begin{equation*} H^{(k+1)}=f(H^{(k)},W) \tag{2}\end{equation*}

where $H^{(0)}=X$ and $H^{(K)}=Z$. The adjacency weight $W_{i,j}$ measures the non-geometric phenotype information similarity between $i$-th subject and $j$-th subject, such as age, gender and APOE, etc. In each network layer, ${f}(H, W)$ is done by graph convolution operation and non-linear activation function.

#### 1) Spectral Graph Convolution Operation

Based on spectral graph theory, the convolution of the graph in the spatial domain is converted into the multiplication in the spectral domain. Assuming a weighted graph ${G} =\{V, E, W\}$, where $V$ represents nodes, $E$ represents edges, and $W$ represents the adjacency matrix of the graph. And its normalized graph Laplacian matrix can be expressed as [36]:

\begin{equation*} L=I_{N} -D^{-\frac {1}{2}}WD^{-\frac {1}{2}} \tag{3}\end{equation*}

where $I_{N}$ is the identity matrix of size $N\times N$ and $D$ is the diagonal degree matrix. An eigen decomposition of the Laplacian matrix, ${L} ={U} \Lambda U^{T}$, gives a set of orthonormal eigenvectors $U= [u_{0}, \ldots, u_{N-1}] \in R^{N\times N}$ with associated real, non-negative eigenvalue diagonal matrix $\Lambda $ = diag([ $\lambda _{0}, \ldots, \lambda _{N-1}$]) $\in R^{N\times N}$. Considering a spatial signal $x$ defined on graph $G$, its Fourier transform is defined as $\hat {\text {x}}=U^{T} x \in R^{N}$, while the inverse transform is given by $\text {x}=U \hat {x}$. Therefore, the graph convolution operation can be defined as:

\begin{equation*} g_{\theta} * x=U\left ({\left ({U^{T} g_{\theta} }\right) \cdot \left ({U^{T} x}\right)}\right) \tag{4}\end{equation*}

where $g_{\theta }$ represents the convolution kernel of graph convolution with the learnable parameters.

#### 2) Graph Edges

Given $M_{l}(m)$ as $l$-th phenotypic information of individual m, the adjacency matrix W is defined as

\begin{equation*} W(m, n)=\sum _{l=1}^{p} \alpha \left ({M_{l}(m), M_{l}(n)}\right) \tag{5}\end{equation*}

where $W$( $m$, $n$) represents the edge weight between individual $m$ and individual $n$, $P$ is the total number of kinds of phenotypic information. And $\alpha $ characterizes the phenotypic information similarity between individuals. When the phenotypic information is categorical type, such as gender, we use the Kronecker delta formula [37] to define $\alpha $. When the phenotypic information is numerical type, such as age and APOE, we define $\alpha $ as a unit step function with a threshold $\theta $. i.e.,

\begin{align*} \alpha \left ({M_{h}(m), M_{h}(m)}\right)=\begin{cases}\displaystyle 1, & a b s\left ({\mathrm {diff}\left ({M_{h}(m), M_{h}(n)}\right)}\right) < \theta \\ \displaystyle 0, & {~\text {else }}\end{cases}\!\!\! \tag{6}\end{align*}

#### 3) Attention Module

**FIGURE 1.**
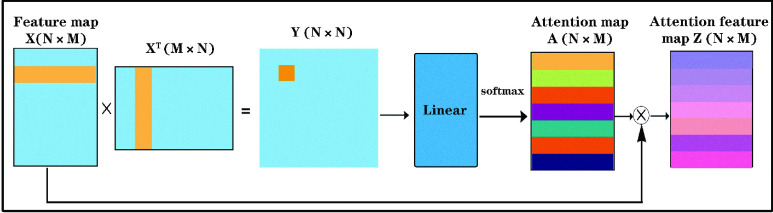
Architecture of attention module. We computed the dot product of every two nodes on the feature map separately, applied a linear layer and a softmax function to obtain the weight map A of ROIs. Finally, we obtained the attention feature map $Z=X\otimes A$.

In this work, we introduced an attention module for highlighting the UNBs generated by the significant ROIs affected by AD. This module can effectively improve the training efficiency and accuracy of the UNB-GCN model by refining the input feature vectors. After obtaining feature vectors $X\{{X}\in R^{N\times M}\}$, we computed the dot product of every two nodes separately, and we reached a feature matrix $Y\{Y\in R^{N\times N}\}$. Then we applied a linear layer and a softmax function to obtain the attention map $A\{A\in R^{N\times M}\}$. Finally, we obtained the attention feature map by computing $Z=X\otimes A$ i.e., $Z = softmax(Linear(X.X^{T}))\otimes X$, where $X\in R^{N\times M}$ represents the original input feature vector map. The attention module was depicted in Fig. 1.

**FIGURE 2.**
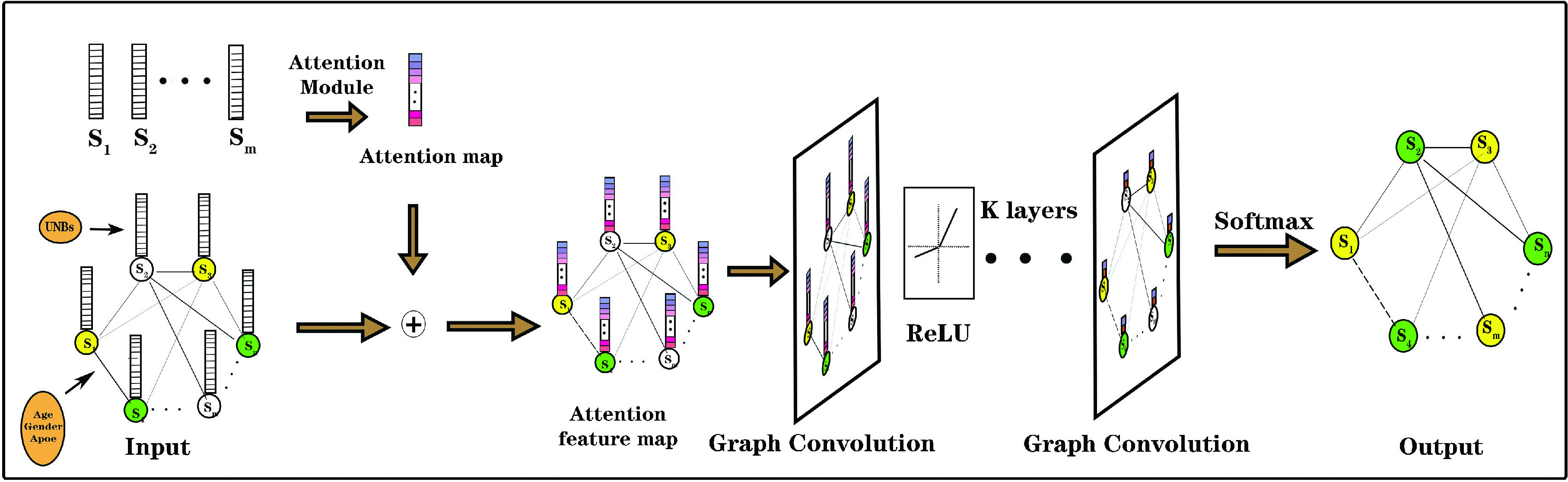
Architecture of our proposed UNB-GCN framework. In this framework, each node represented the subject’s UNB features; the edge weight represented the phenotypic information similarity between two subjects. The model contained an embedded attention module and the GCN with K hidden layers activated by using the Rectified Linear Unit (ReLU) function. The output layer was followed by a softmax activation function.

With the attention module, our UNB-GCN framework was illustrated in Fig. 2. The model consisted of an embedded attention module and a GCN with $K$ hidden layers activated by the rectified linear unit (ReLU) function. The output layer was followed by a softmax activation function. In this framework, each node represented the subject’s UNB features; the edge weight represented the phenotypic information similarity between two subjects. Using the UNB-GCN model constructed by the graph-structured data and the symmetric normalized Laplacian matrix $L$ for graph convolution, we can extract the hidden rich information the refined UNBs through the attention module. By calculating the cross entropy loss function for all labeled nodes, ${f}$( $X$, $W$) on the graph can be trained for a non-labeled node classification. All experiments were run on commodity hardware with 64G RAM and a single 2.8 GHz CPU. The framework adopted was PyTorch 1.0.0 and Python 3.6.1.

## III. Experimental Results

### A. Generation of AD Group Atrophy Pattern

To obtain the AD group atrophy pattern, we analyzed 258 subjects from ADNI database including 127 $\text{A}\beta +$ AD patients and 131 $\text{A}\beta $- CU subjects (Table 1). Because one of the hallmarks of AD is the accumulation of beta-amyloid plaques ( $\text{A}\beta $) in human brains and a positive $\text{A}\beta $ reading is now accepted as ‘dementia due to AD’ together with the presence of clinical symptoms. Using $\text{A}\beta +$ AD subjects to generate AD group atrophy pattern may reflect intrinsic morphological changes induced by AD and also have strong generalization ability on new subjects. Demographic and clinical data were compared using a one-way analysis of variance, and the gender data were analyzed by a $\chi ^{2}$ test. Table 1 indicated that the factors of gender, age and education of the two groups were matched, while the Mini-Mental State Examination (MMSE) was significantly different between these two groups. MMSE is a commonly used cognitive function rating scale [38]. The lower the score of MMSE is, the more severe the dementia is.

**TABLE 1:** Demographic Information of $\text{A}\beta+$ AD Group and $\text{A}\beta$- CU Group

	Gender (M/F)	Age	Education	MMSE
$\text{A}\beta $+ AD	65/62	75.62±7.24	15.99±2.96	23.12±2.02
$\text{A}\beta $- CU	61/70	75.95±5.18	19.08±5.92	29.02±1.08
Inferential Statistics	$\chi ^{2}$= 0.64; ${p}$= 0.78	F = 0.1; ${p}$= 0.76	F = 7.99; ${p}$= 0.051	F = 767.95; ${p} < 0.0001$

### B. Longitudinal Data Analysis

To verify the discrimination power of UNBs, we used 318 longitudinal $\text{A}\beta +$ subjects, including 84 AD, 102 MCI and 132 CU subjects for longitudinal analysis. The demographic characteristic statistics information for the testing subjects was shown in Table 2, such as Clinical Dementia Rating Sum of Boxes (CDR-SB) [39], AD Assessment Scale-Cognitive Subscale (ADAS-Cog11) [40] and MMSE scores. CDR-SB is an important means to evaluate the stage and severity of AD in longitudinal studies and clinical diagnosis. And it is used for grading and follow-up of dementia severity. Usually the higher the score is, the more severe the dementia. ADAS-Cog11 includes orientation, language, structure, use of ideas, immediate word recall and word recognition. It follows the same trend as the CDR-SB, the higher the score, the more severe the dementia. All subjects underwent two tests, including the baseline test and a 24-months test.

**TABLE 2:** Demographic Information of Subjects in Three Clinical Groups

	Gender (M/F)	Age	CDR-SB	ADAS- Cog11	MMSE
AD baseline	42/42	74.37±8.56	4.18±1.70	17.64± 6.25	22.67±1.87
AD m24		76.65±8.96	6.60±3.50	24.37± 11.38	19.19±5.37
MCI baseline	56/46	72.55±7.06	1.57±1.05	10.72± 4.24	28.13±1.56
MCI m24		74.73±7.02	2.52±2.15	12.22± 5.75	26.27±3.05
CU baseline	62/70	75.57±4.34	0.00±0.00	5.74± 2.77	29.24±0.84
CU m24		77.81±4.35	0.08±0.23	5.35± 2.92	29.00±1.07

### C. Group Difference Study

Based on the obtained ROIs, using the AD group atrophy degree and the individual atrophy degree, we computed the UNBs of 318 longitudinal $\text{A}\beta +$ subjects via (1), including 84 AD, 102 MCI and 132 CU subjects for longitudinal analysis. And we studied the total changes of the UNBs and the left hemisphere cortical volume for the longitudinal subjects within a period of 24 months. To adjust for individual differences in head size, the volume of each ROI was adjusted by the intracranial vault volume (ICV) of each ubject (volume/ICV). The statistical comparison results of different longitudinal groups of UNBs and volume measures were shown in Table 3. The $p$-value results of different longitudinal groups were computed by two-sided paired $t$-tests. The effect sizes of different longitudinal groups were computed by paired Cohen’s $d$ measure [41].

**TABLE 3:** The Statistical Comparison Results of Different Longitudinal Groups Based on the UNBS and Volume Measures

Longitudinal group (bl vs. m24)	AD	MCI	CU
$p$-value of UNB	8.21e-09	1.12e-05	1.73e-04
Effect Size of UNB	0.63	0.37	0.26
$p$-value of Volume	1.29e-08	2.08e-04	8.81e-03
Effect Size of Volume	0.56	0.32	0.19

For the longitudinal $\text{A}\beta +$ AD, $\text{A}\beta +$ MCI and $\text{A}\beta +$ CU, the $p$-values and effect sizes for the mean differences of the total UNB were 8.21e-09 and 0.63, 1.12e-05 and 0.37, 1.73e-04 and 0.26, respectively. For the total volume measures, the $p$-values and effect sizes of the longitudinal $\text{A}\beta +$ AD, $\text{A}\beta +$ MCI and $\text{A}\beta +$ CU were 1.29e-08 and 0.56, 2.08e-04 and 0.32, 8.81e-03 and 0.19, respectively. The results indicated that the UNBs might have stronger perception ability to the morphological changes of the cerebral cortex than the volume measures.

### D. Minimum Sample Size Estimation

**FIGURE 3.**
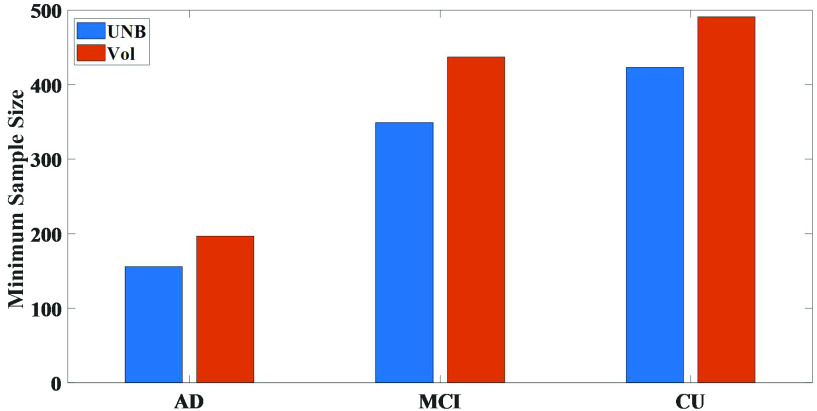
The minimum sample size comparisons between UNBs and volume measures for longitudinal $\text{A}\beta +$ AD, MCI and CU groups.

To assess the statistical power of the obtained UNB, we used the minimum sample size estimation:

\begin{equation*} N=C\frac {\sigma ^{2}}{(m-b)^{2}} \tag{7}\end{equation*}

where $\sigma $ denotes the standard deviation of the biomarkers changes, $m$ and $b$ refer to the mean value of the total UNBs at the 24-months test and the baseline test of the longitudinal data. $C$ is a constant. Using Eq. (7), we estimated the minimum sample sizes of the total UNBs and the total left hemisphere volume measures. As shown in Fig. 3, the minimum sample sizes of the total UNBs of the longitudinal $\text{A}\beta +$ AD, $\text{A}\beta +$ MCI and $\text{A}\beta +$ CU groups were 156, 349 and 423, respectively. For the total volume measures, the minimum sample sizes of the longitudinal $\text{A}\beta +$ AD, $\text{A}\beta +$ MCI and $\text{A}\beta +$ CU groups were 197, 437 and 491.

Regardless of whether it was based on the UNBs or volume measures, the minimum sample sizes of longitudinal $\text{A}\beta +$ AD group was smallest, followed by the longitudinal $\text{A}\beta +$ MCI group, and the minimum sample sizes of longitudinal $\text{A}\beta +$ CU group was largest. It indicated that the morphological changes in the $\text{A}\beta +$ AD group were relatively largest, followed by $\text{A}\beta +$ MCI group, and the morphological changes in $\text{A}\beta +$ CU group were relatively smallest between the baseline and the 24-months follow-up. Meanwhile, the results showed that the minimum sample sizes from the volume measures for different longitudinal groups were larger than our UNBs, indicating that the UNBs might have detected the essential morphological changes induced by AD better than the volume measures and could sensitively identify the degree of abnormal cortical morphological changes caused by neurodegenerative diseases.

### E. Correlation Analysis Between UNBs, Volume Measures and Clinical Rating Scores

In this section, we verified whether the total changes of UNBs and cortical volume measures were correlated with the total changes of clinical rating scores, such as CDR-SB, ADAS-Cog11 and MMSE scores. Then we calculated the change rates ( $R_{f}$) of UNBs, volume measures and clinical rating scores over a period of time by the following equation:

\begin{equation*} R_{f}=\frac {f_{second} -f_{first}}{f_{first}} \tag{8}\end{equation*}

where $f_{\text {first}}$ and $f_{\text {second}}$ represent the total values of UNBs and volume measures at the baseline test and the 24-months test. We used the same $\text{A}\beta +$ longitudinal subjects (84 AD, 102 MCI and 132 CU subjects in Section III.B). We explored the correlation analysis between the $R_{f}$ values of UNBs, cortical volume and the $R_{f}$ values of CDR-SB, MMSE, and ADAS-Cog11 measures with the Pearson parametric test [42], to understand which variables were correlated with UNBs in our dataset. The correlation results, i.e., correlation coefficients (CC), and correlation significance for CC between the $R_{f}$ values of UNBs, volume measures and the $R_{f}$ values of CDR-SB, MMSE, and ADAS-Cog11 measures, were shown in Table 4.

**TABLE 4:** The Correlation Analysis Results Between UNBs, Volume Measures and CDR-SB, ADAS-Cog11 and MMSE for Different $\text{A}\beta+$ Longitudinal Groups

Group	Variables	CC	p-value of CC
AD	UNB vs. CDR-SB	0.43	5.76e $-07\ast \ast $
	UNB vs. ADAS-Cog11	0.40	3.42e $-06\ast \ast $
	UNB vs. MMSE	−0.47	8.43e $-07\ast \ast $
	Vol vs. CDR-SB	−0.35	1.02e $-07\ast \ast $
	Vol vs. ADAS-Cog11	−0.38	2.56e $-06\ast \ast $
	Vol vs. MMSE	0.43	4.64e $-06\ast \ast $
MCI	UNB vs. CDR-SB	0.39	7.01e $-05\ast $
	UNB vs. ADAS-Cog11	0.32	4.25e $-03\ast $
	UNB vs. MMSE	−0.41	3.19e $-04\ast $
	Vol vs. CDR-SB	−0.30	9.45e $-04\ast $
	Vol vs. ADAS-Cog11	−0.31	3.73e $-04\ast $
	Vol vs. MMSE	0.37	1.34e $-03\ast $
CU	UNB vs. CDR-SB	0.10	0.03
	UNB vs. ADAS-Cog11	0.05	0.43
	UNB vs. MMSE	−0.13	3.29e $-03\ast $
	Vol vs. CDR-SB	−0.09	0.04
	Vol vs. ADAS-Cog11	−0.06	0.21
	Vol vs. MMSE	0.11	0.01

The results in Table 4 showed that there were moderate correlations between the $R_{f}$ values of UNBs and the $R_{f}$ values of CDR-SB, ADAS-Cog11 and MMSE scores for $\text{A}\beta +$ longitudinal AD and MCI groups. This was likely due to that the proposed UNBs well match with clinical outcomes within longitudinal $\text{A}\beta +$ AD and MCI groups. However, the $R_{f}$ values of UNBs had weak correlations with the $R_{f}$ values of the CDR-SB, ADAS-Cog11 and MMSE scores for longitudinal $\text{A}\beta +$ CU group. A reasonable reason might be that the morphological changes induced by AD occurred before the cognitive decline in the non-destructive cognitive stage, as previously proposed in the literature [43], [44]. In addition, the correlation between the $R_{f}$ values of UNBs and the $R_{f} $ values of CDR-SB, ADAS-Cog11 and MMSE scores were better than those of volume measures for the all $\text{A}\beta +$ longitudinal groups. In conclusion, UNB, as a measure to compare abnormal morphological patterns of individuals with abnormal morphological patterns of AD group, may better describe the morphological changes of cerebral cortex induced by AD.

### F. Setting

In our experiments, we evaluated the effectiveness of UNB-GCN on the ADNI databases. We used the grid search method and 10-fold cross-validation method to find the optimal parameter combinations [45]. Specifically, we randomly selected 90% of labeled subjects for training and the last 10% for testing. The random splits were repeated 10 times to reduce random errors. Finally, the best combination was selected according to the cross-validation scores. Parameters details are as below: dropout rate was 0.3, the learning rate was 0.01, the number of epochs was 200, the quantitative phenotypic measures $\theta $ was 2, the hidden layers K was 3. Using the above setting, we carried out comprehensive experiments to demonstrate the performance of the UNB-GCN model.

### G. Classification Comparison Between UNB-GCN and VOL-GCN

**FIGURE 4.**
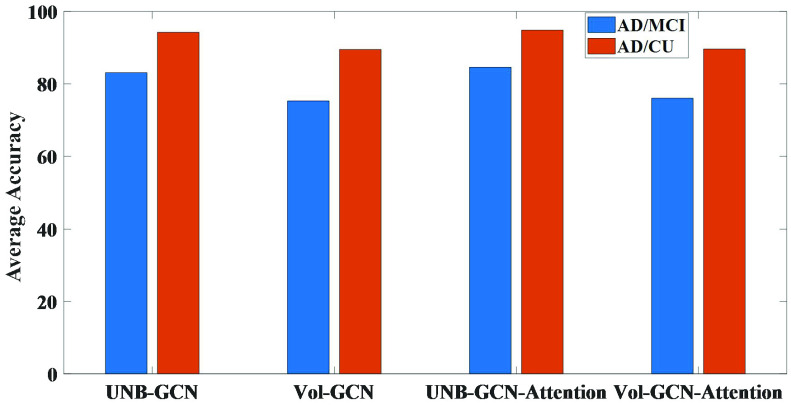
The performances for UNB-GCN and Vol-GCN classification models with and without attention module.

In this section, we used the $\text{A}\beta +$ longitudinal subjects at 24-months test (84 AD, 102 MCI and 132 CU subjects in Section III.B). We took UNBs and volume measures as the biomarker inputs and constructed the UNB-GCN and the Vol-GCN classification models, respectively. To further evaluate the effectiveness of the embedded attention model, we also evaluated the performance for the Vol-GCN and UNB-GCN with and without the attention module, respectively. The results for the discrimination abilities of the different classification models were shown in Fig. 4.

When UNBs were used as the biomarker, the average accuracy was 94.25% for AD vs. CU and 83.11% for AD vs. MCI. When volume measures were used as biomarkers, the average accuracy was 89.50% for AD vs. CU and 75.28% for AD vs. MCI. The results showed that our proposed UNB biomarker had higher classification power than volume measures.

On the other hand, when we used UNB-GCN model with attention module, the average accuracy was 94.84% for AD vs. CU and 84.61% for AD vs. MCI. When we used Vol-GCN model with attention module, the average accuracy was 89.64% for AD vs. CU and 76.04% for AD vs. MCI. The results showed that the embedded attention module may slightly improve the classification performance of the GCN network through highlighting the main impact of AD on the cerebral cortex. Among them, the classification performance of AD vs. MCI was a little improved than that of AD vs. CU. This may be due to the fact that there are some stable significant atrophy patterns for some specific ROIs between MCI and AD groups, which can be learned in the attention model.

### H. Comparisons With some Machine Learning Algorithm

**FIGURE 5.**
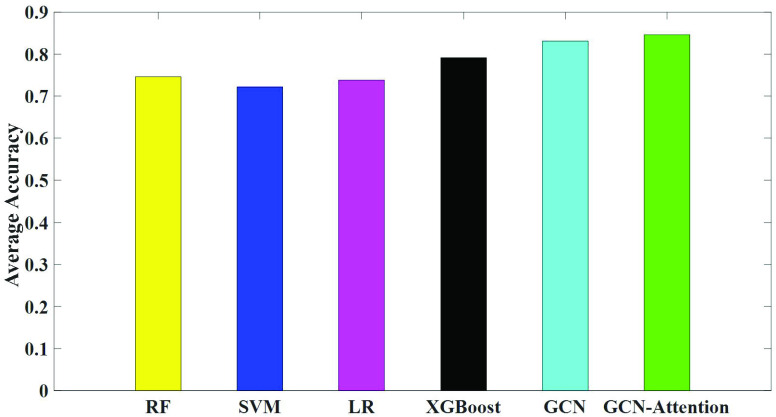
The classification performances of various classification models.

We further compared the classification performance of our UNB-GCN model with various traditional classification models. Using the UNBs as the input, we compared the classification performance of AD and MCI. The traditional classification models included random forest classifier (RF) [46], support vector machine (SVM),logistic regression (LR) [47] and Extreme Gradient Boosting (XGBoost) [48]. The parameters of RF were: The number of trees was 50, and the maximum depth was 25. We used the L_2_ penalty and one-vs-rest (OVR). The parameters of SVM were: The kernel was ‘gaussian radial basis function kernel’, the penalty factor C was 10, regularization parameter was 0.1 and the gamma was 0.05. For the XGBoost, we used the tree-based models, estimators number was 50, min_child_weight was 6, learning rate was 0.1, max_depth was 5. We used the squared L_2_ penalty. And the result was shown in Fig. 5.

Overall, the results showed that SVM model had the worst performance with an accuracy of 72.2%, followed by LR model (73.8% accuracy). And the classification accuracy of the RF classifier is slightly improved to 74.6%. The classification accuracy of the XGBoost is slightly improved to 79.2%. Compared with other methods, the classification performance of GCN was greatly improved, with an average accuracy of 83.11%. The average accuracy of our proposed GCN model with attention module was 84.61%. The results showed that our GCN model outperforms the traditional machine learning classification models.

### I. Comparisons With Some Deep-Learning Based Models

In order to compare the effectiveness of the proposed method on the diagnostic task of AD, we compared the diagnostic accuracy of UNB-GCN model with existing methods (e.g., CNN [49], GCN [50], GAT [51]) on the same dataset in Section III.G. The diagnostic accuracy (ACC), sensitivity (SEN) and specificity (SPE) of the above methods were compared with those of UNB-GCN in AD/NC diagnostic tasks. The performances on AD classification achieved by our method and some deep learning methods on the test set from ADNI were shown in Table 5. And the confusion matrix was shown in Table 6.

**TABLE 5:** Comparison of Our Method With Some Deep-Learning Based Models

Method	ACC	SEN	SPE	AUC
UNB-GCN	94.25%	98.39%	89.13%	95.30%
CNN	92.13%	95.28%	87.64%	92.87%
GAT	93.52%	96.83%	88.89%	93.55%
GCN	90.28%	93.02%	86.21%	90.80%

**TABLE 6:** Confusion Matrix Comparison of Our Method With Some Deep-Learning Based Models

Method	TP	FP	FN	TN
UNB-GCN	122	10	2	82
CNN	121	11	6	78
GAT	122	10	4	80
GCN	120	12	9	75

Our method reached better results on all four metrics (i.e., ACC = 94.25%, SEN = 98.39%, SPE = 89.13%, and AUC = 95.30%) in AD vs. CU classification. We set the epochs as 200, the learning rate as 0.01, dropout rate: 0.3. CNN model achieved ACC = 92.13%, SEN = 95.28%, SPE = 87.64%, and AUC = 92.87%. GAT model achieved ACC = 93.52%, SEN = 96.83%, SPE = 88.89%, and AUC = 93.55%. GCN model achieved ACC = 90.28%, SEN = 93.02%, SPE = 86.21%, and AUC = 90.80%. GAT models perform better than GCN models. These results are consistent with findings in previous works [48], [49]. In contrast, our proposed method achieved accuracy 0.7% higher than the best results using the above methods. The results shown in Table 6 indicated that True Positive (TP) of UNB-GCN model and GAT model were better than other models. And the True Negative (TN) of the UNB-GCN model was better than the others. The False Negative (FN) and the False Positive (FP) of the UNB-GCN model were smaller than the other models.

### J. Validating the Model on the Validation Set

In this section, we created an external validation set from ADNI to further evaluate the performance of our model. we used 128 $\text{A}\beta +$ subjects which were at 24-months test, including 32 AD, 46 MCI and 50 CU subjects. The demographic characteristic statistics information was shown in Table 7.

**TABLE 7:** Demographic Information of Subjects in Three Clinical Groups

	Gender (M/F)	Age	CDR-SB	ADAS-Cog11	MMSE
AD	15/17	75.42±6.86	5.75±2.18	20.26±5.36	20.42±3.43
MCI	26/20	73.11±6.34	1.86±1.85	10.12±4.75	27.93±2.43
CU	24/26	76.05±4.87	0.00±0.00	6.46±2.24	29.55±0.97

We firstly generate new UNBs with our proposed UNB algorithm for all the new subjects from ADNI according to the Eq. (1). Then we validated the trained UNB-GCN model in Section III.F. The validation set achieved ACC = 93.90%, SEN = 90.91%, SPE = 95.92%, and AUC = 95.85% in AD vs. CU classification. Our model achieved ACC = 82.05%, SEN = 76.47%, SPE = 86.36%, and AUC = 86.60% in AD vs. MCI classification. And the confusion matrix was shown in Table 8.

**TABLE 8:** Confusion Matrix of Our Method on the Validation Set

Method	TP	FP	FN	TN
AD vs. CU	30	2	3	47
AD vs. MCI	26	6	8	38

### K. Comparisons to the Related Prior Works

In this section, we further compare our UNB-GCN method with other different competing methods in the corresponding papers. Poloni et al. proposed a two-level classification framework, landmark- and image-based [52]. They classified 209 AD patients and 302 CU subjects. The ACC was 89.24%. They classified 209 AD patients and 251 MCI patients. The ACC was 69.8%. Jiao et al. proposed a multi-modal feature selection algorithm using feature correlation and feature structure fusion [53]. The accuracy of CU subjects versus AD patients achieved 91.85%. Wu et al. proposed an entropy-based measure of causality brain networks on the basis of the rs-fMRI data to classify AD and CU [54]. The ACC was 89.83%. Jiang et al. proposed a hierarchical GCN framework (hi-GCN) to learn the graph feature embedding while considering the network topology information and subject’s association at the same time [55]. The accuracy of MCI subjects versus AD patients achieved 78.5%. Table 9 showed the comparison results. We can observe that our proposed method had achieved promising performance. Hence, our model had a good application prospect in classification tasks.

**TABLE 9:** Algorithm Comparison With the Related Works

References	Classifier	Data	ACC
Poloni 2022 [52]	A two-level classification framework, landmark- and image-based	209 AD vs. 302 CU	89.24%
		209 AD vs. 251 MCI	69.8%
Jiao 2022 [53]	Multi-modal feature selection	69 AD vs. 73 CU	91.85%
Wu 2021 [54]	Combining the Sample entropy with dynamic causality analysis	29 AD vs.30 CU	89.83%
Hao 2020 [55]	Hierarchical graph convolution network	34 AD vs. 99 MCI	78.5%
Ours	UNB-GCN	32 AD vs.50 CU	93.90%
		32 AD vs. 46 MCI	82.05%

## IV. Discussion

We proposed the UNB-GCN classification framework that can effectively discriminate between MCI subjects and AD patients. With the reliability and sensitivity of UNB to AD-induced cerebral cortical morphological changes and the enhancement effect of attention module on the UNB features, our proposed classification model can make full use of the morphological features of individuals and the correlation between individual phenotypic information for training and classification, thereby improve the computational efficiency and accuracy of the classification. The experimental results indicated that our proposed UNB measures were superior to the conventional volume measures, and the UNB-GCN framework combined with attention module could effectively improve the classification performance.

### A. Influence of the General Linear Model

**FIGURE 6.**
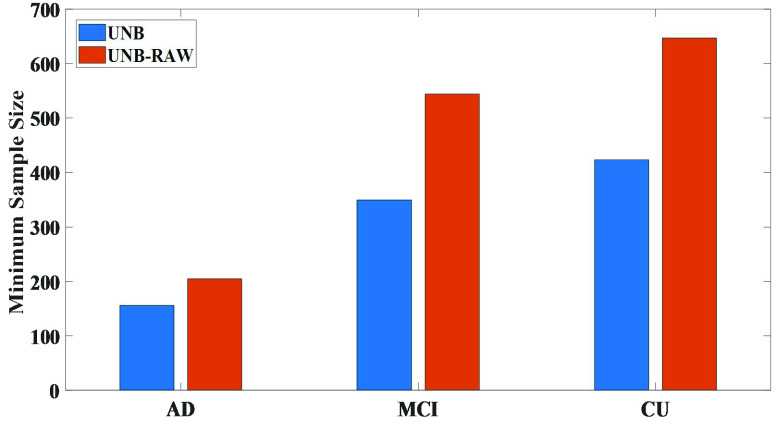
The minimum sample sizes of the UNB and UNB-RAW.

In this paper, we used the general linear model to obtain intrinsic group morphological structure among the same group excluding the influence of individual differences of age and gender. In order to explore the influence of these factors on UNBs, we directly used the raw thickness features without the general linear model processing. We selected same subjects (127 $\text{A}\beta +$ AD patients and 131 $\text{A}\beta $- CU subjects in Section III.A). We followed the method proposed in Section II.D to calculate the new UNBs based on the raw thickness information and we called the new UNBs as UNB-RAWs. With Eq. (7), we estimated the minimum sample sizes of the total UNB-RAWs. The results were shown in Fig. 6.

For the UNB-RAWs, the minimum sample sizes of the longitudinal $\text{A}\beta +$ AD, $\text{A}\beta +$ MCI and $\text{A}\beta +$ CU groups were 205, 544 and 647, respectively. For the UNBs, the minimum sample sizes of the longitudinal $\text{A}\beta +$ AD, $\text{A}\beta +$ MCI and $\text{A}\beta +$ CU groups were 156, 349 and 423, respectively. The experiments showed that the general linear model can eliminate the influence of individual differences of age and gender. It enabled us to extract intrinsic morphological changes of cortical surface induced by AD. Using general linear model, we could improve the stability of UNB and improve statistical discrimination power.

### B. ROC Analysis

To further access the statistical discrimination power of the UNBs, we used ROC method to compare the discrimination performance of the total UNBs and the total volume measures in distinguishing $\text{A}\beta +$ AD and $\text{A}\beta +$ CU subjects. We used the $\text{A}\beta +$ longitudinal subjects (84 AD and 132 CU subjects in Section III.A). Then we applied the ROC curve to analyze the statistical discriminative power of the total UNBs and the total volume measures.

**FIGURE 7.**
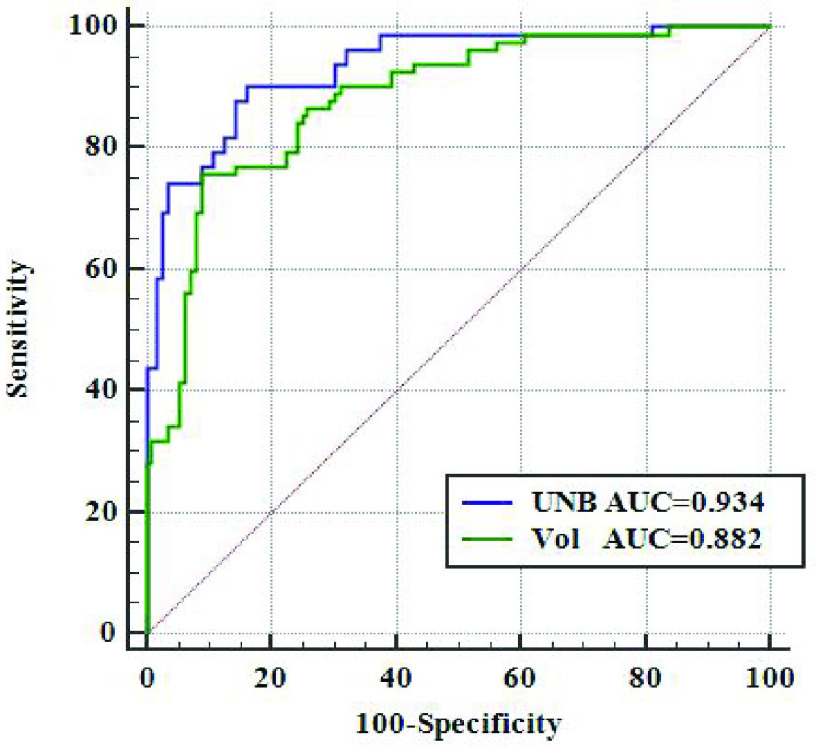
The ROC results of the UNBs and the volume measures.

The results in Fig. 7 showed that the AUC, 95% confidence interval (CI) of AUC for classifying the $\text{A}\beta +$ AD and $\text{A}\beta +$ CU subjects are 0.934 and [0.890, 0.965] for the UNBs, 0.882 and [0.848, 0.904] for the volume measures, respectively. This indicated that the UNBs may better characterize the degree of influence of AD on individual morphology than the volume measures.

### C. Influence of the Phenotypic Measures

**FIGURE 8.**
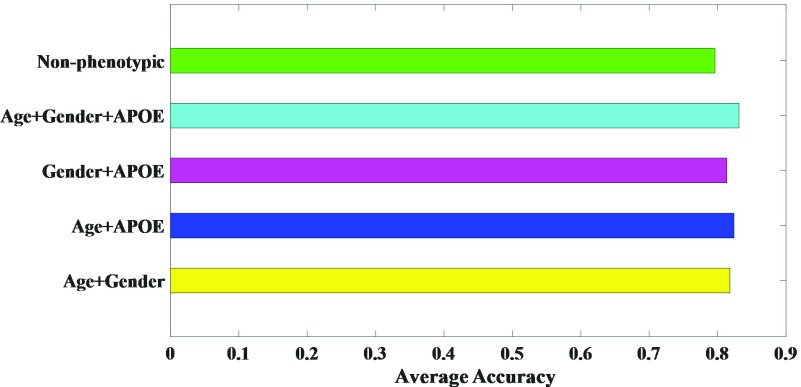
Classification results of different phenotypic information.

To further improve the classification performance of our UNB-GCN model, we incorporated phenotypic information to further optimize the graph structure. In this section, we evaluated the performance of the model under different phenotype graph configurations for AD vs. MCI. Phenotypic information included age, gender and APOE genetic information. The results of multiple different graph resulted measured using gender, age or APOE genetic information was presented in Fig. 8.

The experiments verified whether the classification performance would change with the combination change of phenotypic information. Among them, the combination of age and APOE genetic information showed the largest performance with an average accuracy of 82.4%. This was followed by a combination of age and gender, with an average accuracy of 81.8%. And the combination of gender and APOE genetic information had an average accuracy of 81.35%. The average accuracy of the classification was 79.6% without combining phenotypic information. The results showed that the classification performance of our GCN model was improved when we incorporated age, gender and APOE genetic information into the construction of the weights of edges in the graph structure. The average accuracy of the classification was 83.11%.

### D. Attention Module for ROIs Extraction

**FIGURE 9.**
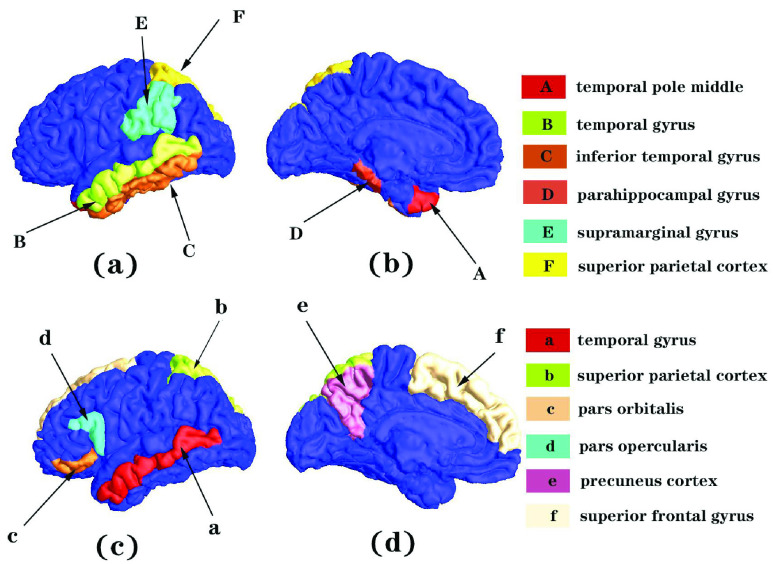
The localization of the significant ROIs. ROIs with normalized attention weights smaller than 0.05 were not displayed. Fig (a) and Fig (b) showed that the GCN-UNB model with an attention module highlights the significant group-difference regions for CU vs. AD. Fig (c) and Fig (d) showed that the GCN-UNB model with an attention module highlights the significant group-difference regions for MCI vs. AD.

In our GCN model, we embedded an attention module that could generate attention maps via forward propagation. The attention map indicated ROIs with significant morphological changes induced by AD, which were normalized to a range of 0-1 for visualization. ROIs with the normalized attention weights smaller than 0.05 were not displayed. As shown in Fig. 9, it illustrated the localization of the significant ROIs.

The results show that the UNB-GCN model with an attention module highlights the significant group-difference regions for CU vs. AD and MCI vs. AD. Fig (a) and Fig (b) showed regions with significant morphological difference between CU and AD groups, including temporal pole middle ( $w$ = 0.1065), temporal gyrus ( $w$ = 0.0815), inferior temporal gyrus ( $w$ = 0.0708), parahippocampal gyrus ( $w$ = 0.0596), supramarginal gyrus ( $w$ = 0.0553) and superior parietal cortex ( $w$ = 0.0508). Fig (c) and Fig (d) showed regions with significant morphological difference between MCI and AD groups, including temporal gyrus ( $w$ = 0.0630), superior parietal cortex ( $w$ = 0.0615), pars orbitalis ( $w$ = 0.0575), pars opercularis ( $w$ = 0.0548), precuneus cortex ( $w$ = 0.0512), superior frontal gyrus ( $w$ = 0.0508). The obtained significant group-difference regions induced by AD in different group comparisons, i.e., CU vs. AD and MCI vs. AD, were consistent with the observations in prior researches [31], [56].

### E. Limitations

We should also note the limitations of this study. First, a relatively small number of subjects were included as the research objects. Besides, we had not tested our GCN model and UNB algorithm in a cohort other than ADNI. Even so, our current results demonstrated the proposed GCN model might improve the classification performance of AD. Third, we used UNBs only to identify AD without combining other types of biomarkers. If we combined these biomarkers with UNBs, the ability to identify AD could be effectively improved. Finally, we need to continue to optimize the network model used in the experiment to improve the robustness of the model classification.

## V. Conclusion and Future Work

In this paper, we proposed a UNB-GCN classification framework which may achieve the better classification performance between MCI subjects and AD patients. Recently, functional connectivity networks constructed from functional magnetic resonance images (f-MRI) have shown great promise for distinguishing patients with neurological diseases from normal controls. In our future work, we will use GCN model to fully exploit the complementary information between multimodalities (MRI, PET, and CSF) to fully extract AD-induced features and ultimately achieve better classification performance.
